# Risk Warning of Neutrophil‐to‐Lymphocyte Ratio for Neurological Recovery in Acute Ischemic Stroke After Thrombolysis: A Retrospective Study

**DOI:** 10.1002/brb3.70373

**Published:** 2025-02-28

**Authors:** Yiran Zhao, Xu Wang, Wenxiu Qin, Shaojing Shi, Min Wang, Jinsheng Zhang, Xin Zou, Junfeng Xu, Jing Li, Xuemin Shi

**Affiliations:** ^1^ Department of Acupuncture and Moxibustion First Teaching Hospital of Tianjin University of Traditional Chinese Medicine Tianjin China; ^2^ Department of Acupuncture and Moxibustion National Clinical Research Center for Chinese Medicine Acupuncture and Moxibustion Tianjin China

**Keywords:** acupuncture comprehensive treatment, acute cerebral infarction, neurological function recovery, NLR reduction values

## Abstract

**Background:**

To investigate the relationship between dynamic changes in the neutrophil‐to‐lymphocyte ratio (NLR) and neurological recovery in noninfected patients with acute cerebral infarction after thrombolysis.

**Methods:**

A retrospective analysis was conducted on 277 patients with stroke thrombolysis. Least absolute shrinkage and selection operator (LASSO) regression was used to identify factors influencing recovery, and 1:1 propensity matching was performed between the groups to compare the changes in the National Institutes of Health Stroke Scale (NIHSS) score, muscle strength, and NLR after treatment. The receiver operating characteristic (ROC) curve was used to determine the cutoff value of NLR reduction after treatment, which serves as a diagnostic criterion for neurologic recovery. The posttreatment NLR reduction values were categorized according to whether they were equal to and greater than the cutoff value. Logistic regression was performed after intergroup matching to analyze the relationship between NLR and NIHSS.

**Results:**

The LASSO regression suggests that increased disease duration and atrial fibrillation are risk factors for neurologic recovery, whereas prolonged treatment duration and increased NLR reduction value are protective factors. Compared with the low‐efficacy group, the high‐efficacy group exhibited significantly lower NIHSS scores, NLR, and upper and lower extremity muscle strength scores after treatment (*p* < 0.05). The NLR reduction value was positively correlated with the NIHSS score reduction value and the change in the NIHSS score reduction rate (*p* < 0.001). The ROC curve and logistic regression indicated that patients with an NLR reduction value of ≥ 0.335 had good recovery.

**Conclusions:**

Higher NLR reduction values indicate an improved neurologic prognosis after treatment.

**Trial registration:**

Clinical Retrospectively Registered Trials: ChiCTR2100045415

## Graphical abstract

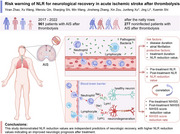

**Abbreviations**
AF: atrial fibrillation
AIS: acute ischemic stroke
DBP: diastolic blood pressure
HT: hemorrhagic transformation
LASSO: least absolute shrinkage and selection operator
LEMSS: lower extremity muscle strength score
NIHSS: National Institutes of Health Stroke Scale
NLR: neutrophil‐to‐lymphocyte ratio
PSM: propensity score matching
ROC: receiver operating characteristic
r‐tPA: recombinant tissue plasminogen activator
SBP: systolic blood pressure
TCM: traditional Chinese medicine
UEMSS: upper extremity muscle strength score

## 1 Introduction

The inflammatory response plays a vital role in all pathophysiological mechanisms of acute ischemic stroke (AIS) (Chamorro and Hallenbeck 2006). In conjunction with cytokines and chemokines released from the ischemic tissue at the affected site, the influx of peripheral circulating leukocytes, particularly neutrophils, is considered a significant contributor to brain damage following ischemia (Dong et al. 2019; Qun et al. 2017; Wu et al. 2013). Numerous studies have highlighted the prognostic relevance of neutrophil‐linked ratios in AIS (Gao et al., 2021; Gong et al., 2021). The neutrophil‐to‐lymphocyte ratio (NLR), a vital biomarker for assessing patient prognosis, is significant in AIS treatment (Sharma et al., 2021; Xue et al., 2017). Moreover, its efficacy has been demonstrated as an indicator for evaluating both short‐ and long‐term patient outcomes (Komurcu et al., 2020; Guo et al., 2016).

Current investigations into NLR in cerebral infarction predominantly focus on static baseline values before thrombolytic therapy, potentially overlooking a comprehensive evaluation of the dynamic fluctuations in patients' conditions throughout the disease course. In this study, we examined the fluctuating trends of NLR values of pretreatment and posttreatment. Furthermore, we explored the relationship between the reduction in these values and the clinical outcomes of noninfected patients who underwent thrombolysis for acute cerebral infarction. We retrospectively analyzed the predictive value of reduced NLR values in neurological function recovery. Additionally, we validated the significance of NLR as a reference marker for cerebral infarction prognosis.

## 2 Materials and Methods

### 2.1 General Information

**FIGURE 1:**
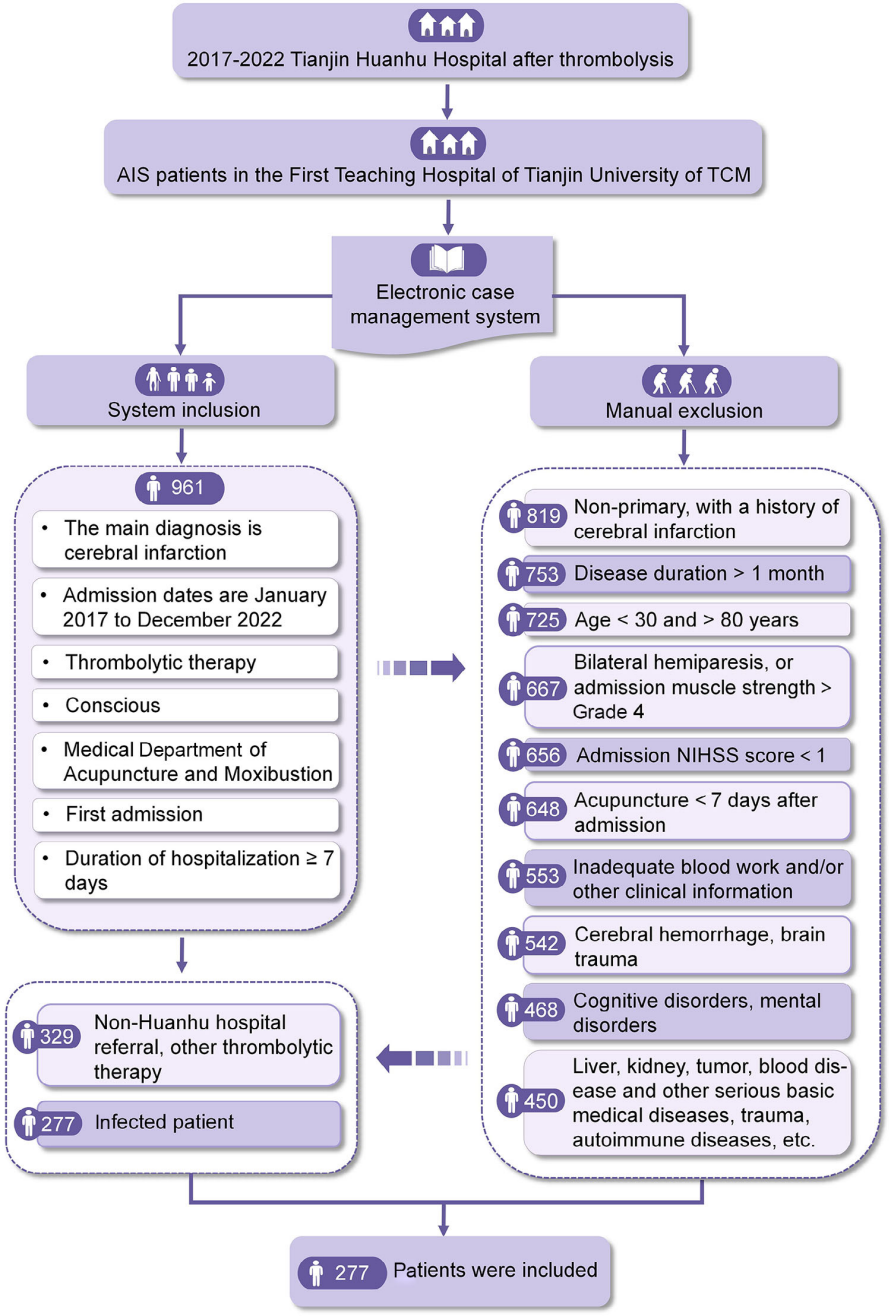
Flowchart of the research subjects. AIS, acute ischemic stroke; NIHSS, National Institutes of Health Stroke Scale; NLR, neutrophil‐to‐lymphocyte ratio; TCM, traditional Chinese medicine.

From January 2017 until December 2022, 277 patients diagnosed with cerebral infarction were relocated to the Department of Acupuncture and Moxibustion at the First Affiliated Hospital of Tianjin University of Traditional Chinese Medicine following intravenous thrombolysis with recombinant tissue plasminogen activator (r‐tPA) at the Tianjin Huanhu Hospital (Figure 1). These patients underwent careful evaluation at our clinic. Among them, there were 208 men and 69 women of an age range of 30–80 years (average: 61.18 ± 9.59 years), and the duration of the disease was ≤ 1 month. The high‐efficacy (*n* = 181) and low‐efficacy (*n* = 96) groups were classified based on whether the reduction rate of the National Institutes of Health Stroke Scale (NIHSS) score was ≥ 18%. This study was approved by the Ethics Committee of the First Teaching Hospital of Tianjin University of Traditional Chinese Medicine (Approval no.: TYLL2020[K]057) and was registered in the Chinese Clinical Trial Registry (Registration no.: ChiCTR2100045415), and an informed consent was granted.

### 2.2 Diagnostic Criteria

#### 2.2.1 Diagnostic Criteria of Chinese Medicine

The relevant diagnostic criteria of the Diagnostic and Efficacy Assessment Criteria for Stroke Disease (for trial implementation), (Ren et al., 1996) as formulated by the Brain Disease Emergency Collaborative Group of the State Administration of Traditional Chinese Medicine (SACTM) in 1996 were met.

#### 2.2.2 Western Medical Diagnostic Criteria

The present diagnostic criteria satisfied the pertinent criteria outlined in the Chinese Guidelines for Diagnosis and Treatment of Acute Ischemic Stroke 2018 (Peng et al., 2018), a publication developed by the Cerebrovascular Disease Group of the Neurology Section of the Chinese Medical Association in 2018.

### 2.3 Inclusion Criteria

(1) Individuals who met both the Chinese and Western criteria for cerebral infarction diagnosis. (2) Individuals experiencing their first stroke episode. (3) Individuals who were administered intravenous thrombolysis with r‐tPA following the occurrence of cerebral infarction, and the duration of the said infarction was < 1 month. (4) Age range 30–80 years. (5) Patients who were conscious and presented with unilateral limb hemiparesis, having muscle strength in the upper and/or lower limbs at ≤ Grade 4. (6) Patients who were admitted to the hospital with an NIHSS score ≥ 1. (7) No prior acupuncture treatment had been administered to any of them before their admission to the study. The duration of acupuncture intervention lasted for at least 7 days following their admission to our hospital. (8) The clinical information of all participants was complete and could be traced back accurately.

### 2.4 Exclusion Criteria

(1) Patients presenting with the combined underlying conditions of severe hepatic, renal, cardiovascular, cerebrovascular, respiratory, gastrointestinal, or endocrine systems that could not be effectively managed clinically or had a previous medical history of hematological disorders or tumors. (2) During the week before their admission or after admission of the patients with infections, traumatic injuries, autoimmune diseases, or those who were receiving medications that could impact their leukocyte count. (3) Patients with apparent cognitive disorders or psychiatric disorders. (4) Absence of neurological deficits in patients with cerebral infarction. (5) Cerebral hemorrhage or traumatic brain injury. (6) Pregnant or breastfeeding women. (7) Patients who were admitted and/or discharged without routine blood testing. (8) Patients who are unable to undergo acupuncture due to diverse factors.

### 2.5 Data Collection

The demographic information of all registered patients was recorded, encompassing gender, age, duration of illness and treatment, as well as the admission indicators such as white blood cell count, fasting blood glucose level, blood pressure, family history of stroke, and medical history of hypertension, diabetes mellitus, coronary artery disease, atrial fibrillation (AF), or hyperlipidemia. Additional information involved data on muscle strength, NIHSS score, neutrophil count, lymphocyte count, and the NLR calculation before and after treatment.

### 2.6 Treatment Method

Both the high‐efficacy and low‐efficacy groups received basic Western medicine treatment in conjunction with the Xingnao Kaiqiao acupuncture treatment.

#### 2.6.1 Basic Treatment

We referred to the Chinese Guidelines for Diagnosis and Treatment of Acute Ischemic Stroke 2018 (Peng et al., 2018) for relevant neurological routine treatment.

#### 2.6.2 Acupuncture Treatment

All patients were treated with the Xingnao Kaiqiao acupuncture method established by the academician Shi (1998), and the acupuncture points were needled once a day during the hospitalization period. The main points of the Xingnao Kaiqiao acupuncture method are Neiguan (PC 6), Renzhong (GV 26), and Sanyinjiao (SP 6), and the supporting acupoints are Jiquan (HT 1), Chize (LU 5), and Weizhong (BL 40).

### 2.7 Observation Indicators

#### 2.7.1 Clinical Efficacy Indicators

##### 2.7.1.1 NIHSS Score

Patients underwent NIHSS assessments before the treatment and at the time of discharge. The NIHSS score was utilized to assess the extent of neurological impairment in individuals with cerebral infarction. The scores ranged from 0 to 42, with higher scores indicating more pronounced neurological deficits. The NIHSS reduction rate was calculated as follows:

NIHSS reduction rate = (NIHSS score at admission − NIHSS score at discharge)/NIHSS score at admission.

Cured: 91%–100% reduction in the NIHSS score.

Significant: 46%–90% reduction in the NIHSS score.

Effective: 18%–45% reduction in the NIHSS score.

Ineffective: decrease or increase in the NIHSS score by ≤ 17%.

Worsening: NIHSS score increases by ≥ 18%.

In this research, the assessment of neurological recuperation was determined based on the rate of reduction in the NIHSS score posttreatment. The subjects were classified into two groups: a high‐effective group (≥ 18% decrease in the NIHSS score) and a low‐effective group (< 18% decrease or increase in the NIHSS score).

##### 2.7.1.2 Lovett Myometry

The muscle strength of the patients before and after treatment was evaluated by using the Lovett unarmed muscle strength examination method, which was scored on a scale of 0–5, with higher scores indicating better muscle strength. The muscle strength of the upper and lower limbs at levels 0–5 was assessed sequentially before and after the treatment, with scores decreasing from 5 to 0. As the score for muscle strength increased, the level of impairment worsened.

#### 2.7.2 Laboratory Indicators

Blood samples were collected mainly through two time points, after admission and the day before discharge; venous blood was collected after 10 h of fasting. Complete blood counts and fasting blood glucose were analyzed using an automatic blood analyzer, and the NLR was calculated.

### 2.8 Data Quality Control

The data collection of this study was mainly completed by three types of researchers, with separate individuals being responsible for data input, review, and statistical analysis, and blinding was implemented among all three. The clinical profile data of the eligible patients were extracted from the intelligent platform and imported into an Excel spreadsheet to establish a database. To ensure the data's authenticity and objectivity, the researchers must trace the original patient data in the medical record system, verify the exported contents for each study, and ultimately conduct statistical analysis.

### 2.9 Statistical Processing

#### 2.9.1 Propensity Matching

Propensity matching can alleviate sample size disparities among groups and minimize the influence of confounding variables. Hence, there is a need to set the corresponding caliper value, which is also known as the tolerance value. For 1:1, the matching tolerance values are mostly 0.02–0.03; the larger the value, the easier it is to match, albeit it is more likely to increase the imbalance between the groups. Moreover, the more stringent the match is, the more likely it is to lead to the deletion of effective patient data, which reduces the effectiveness of the statistics. In this study, by sequentially debugging the intergroup pairings within the tolerance values, the final tolerance value for the 1:1 matching set for the NIHSS subtraction rate high‐efficacy and low‐efficacy groups was 0.03, and the tolerance value for the 1:1 matching of the NLR difference ≥ 0.335 and < 0.335 groups was 0.03.

#### 2.9.2 Processing of Data

**FIGURE 2:**
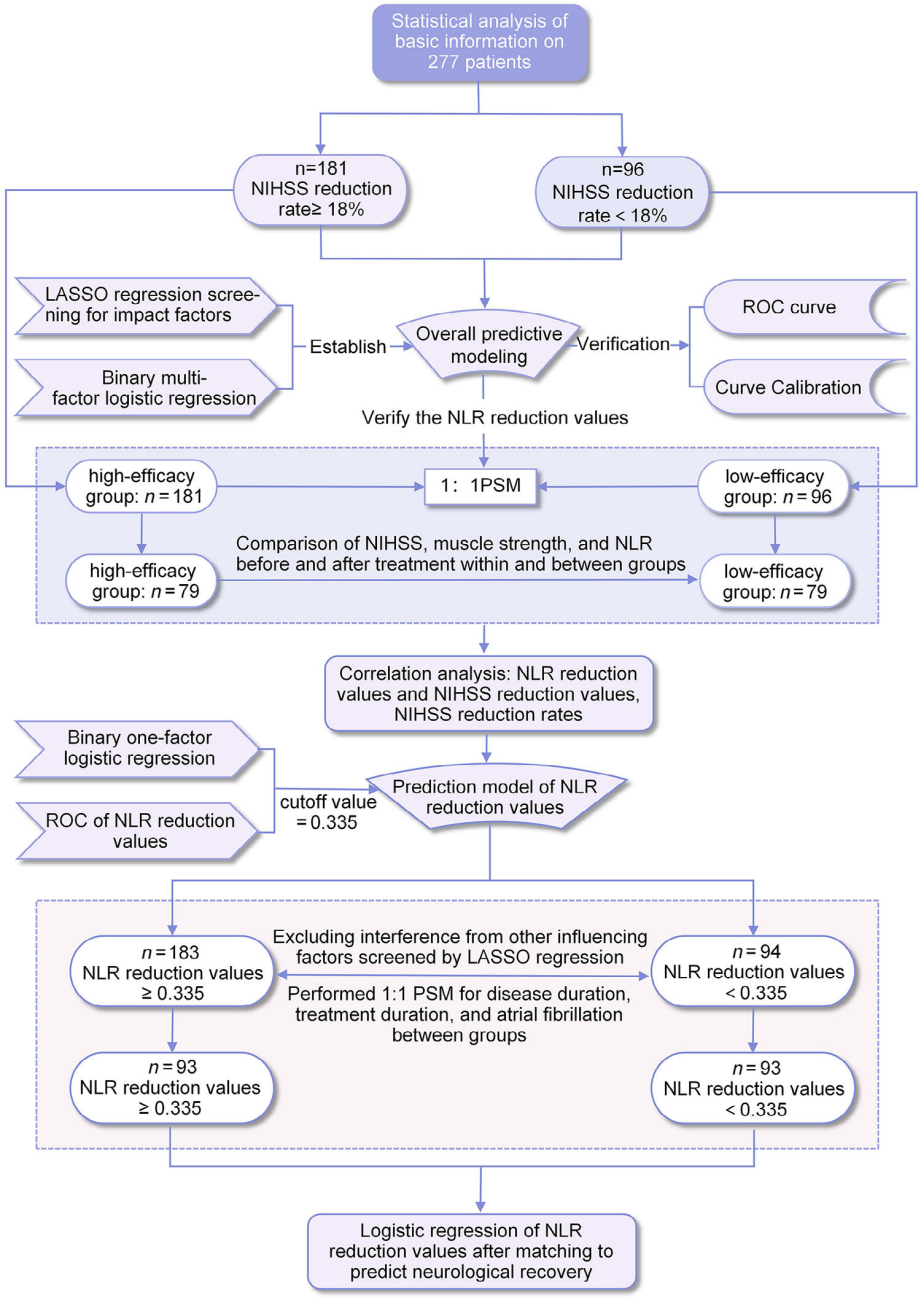
Flowchart of the study design. LASSO, least absolute shrinkage and selection operator regression; NIHSS, National Institutes of Health Stroke Scale; NLR, neutrophil‐to‐lymphocyte ratio; ROC, receiver operating characteristic curve.

All data were entered into the computer, and SPSS 26.0 statistical software was applied for statistical processing. Least absolute shrinkage and selection operator (LASSO) regression with R4.3.0 was performed to screen for predictive influence factors, and the rms package was used to construct the logistic regression model and calibration graphs, while the pROC package was used to assess the extent of the predictive model by calculating the area under the receiver operating characteristic (ROC) curve. The measurement data were tested for normality and homogeneity of variance. The comparison between the groups meeting the normal distribution and homogeneous variance was performed by independent sample *t*‐test, with the mean (SD) indicated. The Mann–Whitney *U* test was performed for comparison between the groups that did not conform to the normal distribution and/or uneven variance, while the median (IQR) was used for the expression. The Wilcoxon signed‐rank test was applied for within‐group comparisons; *χ* ^2^ test was applied for count data expressed as the number of cases and percentage (%). The correlation between the NLR and NIHSS scores was analyzed through bivariate analysis; the predictive value of the reduced value of NLR on neurological recovery before and after treatment was analyzed by applying the ROC curve, and the effect of the reduced value of NLR on the neurological recovery of cerebral infarction was analyzed by using univariate binary logistic regression. All differences were considered statistically significant at *p* < 0.05. The study design flowchart is detailed in Figure 2.

## 3 Results

### 3.1 Predictive Modeling of Neurological Recovery

The study used various continuous and dichotomous variables as independent factors to evaluate their influence on neurological function improvement, which was measured by a ≥ 18% reduction in the NIHSS score. Continuous variables included age, disease duration, treatment duration, admission NIHSS score, admission NLR, discharge NLR, NLR reduction value, admission upper and lower extremity muscle strength score, admission blood counts (leukocyte, neutrophil, and lymphocyte counts), fasting blood glucose levels, and blood pressure. Dichotomous variables included sex, smoking, alcohol consumption, family history of stroke, medical history (hypertension, diabetes mellitus, coronary heart disease, myocardial infarction, AF, and hyperlipidemia), and other clinical factors. LASSO regression analysis was used to reduce variable dimensionality and screen predictors. Logistic regression analysis was employed for model development and curve calibration, and the area under the ROC curve was used to assess the predictive model's performance.

#### 3.1.1 Descriptive Analysis of the Influencing Factors Related to NIHSS Scores

The basic characteristics of the influences associated with NIHSS scores are described as follows (Table 1).

**TABLE 1:** Descriptive analysis of factors influencing the NIHSS scores.

Influencing factors	≥ 18% (*n *= 181)	< 18% (*n* = 96)	*X* ^2^/*Z*/*t*	*p*
Sex, *n* (%)				
Female	48 (26.5)	21 (21.9)	0.723^a^	0.395
Male	133 (73.5)	75 (78.1)		
Age (year)	63 (55, 67.5)	61.5 (55, 68.75)	−0.382^b^	0.703
Disease duration	10 (8, 13)	10.5 (9, 14)	−2.359^b^	0.018^&^
Treatment duration	19 (14, 26.5)	14 (14, 18)	−4.354^b^	0.000*
Treatment duration > 14 days	117 (64.6)	42 (43.8)	11.196^a^	0.001^#^
Admission NIHSS	7 (5, 10)	6 (4, 9)	−2.459^b^	0.014^&^
Admission UEMSS	2 (1, 5)	2 (1, 3)	−1.119^b^	0.263
Admission LEMSS	2 (1, 3)	2 (1, 2)	−2.642^b^	0.008^#^
Admission NLR	3.22 (2.41, 4.60)	2.68 (2.02, 3.57)	−3.826^b^	0.000*
Discharge NLR	2.14 (1.62, 2.78)	2.46 (1.75, 3.29)	−1.850^b^	0.064
NLR reduction values	1.00 (0.36, 1.96)	0.29 (−0.26, 1.00)	−5.723^b^	0.000*
Leukocyte count	7.51 (1.86)	7.12 (1.70)	−1.754^c^	0.081
Neutrophil count	5.09 (3.93, 6.17)	4.60 (3.64, 5.75)	−2.256^b^	0.024^&^
Lymphocyte count	1.53 (1.15, 1.89)	1.62 (1.41, 2.08)	−2.304^b^	0.021^&^
Fasting blood glucose	6.03 (4.98, 7.55)	5.66 (5.00, 7.24)	−0.389^b^	0.698
SBP (mmHg)	135 (125, 146)	135 (124, 145)	−0.584^b^	0.559
DBP (mmHg)	86 (78, 93.5)	84 (77.25, 94.50)	−0.422^b^	0.673
Family stroke history	37 (20.4)	17 (17.7)	0.299^a^	0.585
Smoking history	103 (56.9)	52 (54.2)	0.191^a^	0.662
Drinking history	90 (49.7)	50 (52.1)	0.140^a^	0.709
Hypertension	129 (71.3)	73 (76.0)	0.723^a^	0.395
Diabetes	63 (34.8)	32 (33.3)	0.060^a^	0.806
Coronary heart disease	49 (27.1)	29 (30.2)	0.305^a^	0.581
Myocardial infarction	8 (4.4)	2 (2.1)	0.427^a^	0.513
Atrial fibrillation	15 (8.3)	19 (19.8)	7.710^a^	0.005^#^
Hyperlipidemia	12 (6.6)	4 (4.2)	0.699^a^	0.403

*Note*: Statistically significant covariates: for between‐group comparisons, *p *> 0.05 suggests baseline concordance. Statistically significant covariates: for between‐group comparisons, *p* > 0.05 suggests baseline concordance, *p < 0.001, ^#^ *p* < 0.01, ^&^ *p* < 0.05.

Abbreviations: DBP, diastolic blood pressure; IQR, interquartile range; LEMSS, lower extremity muscle strength score; NIHSS, National Institutes of Health Stroke Scale; NLR, neutrophil‐to‐lymphocyte ratio; SBP, systolic blood pressure; SD, standard deviation; UEMSS, upper extremity muscle strength score.

^a^ Data are *n* (%), *χ* ^2^ test.

^b^ Median (IQR), Mann–Whitney *U* test.

^c^ Mean (SD), two‐sample *t*‐test.

#### 3.1.2 Screening for Factors Affecting Improvement in NIHSS Scores

**FIGURE 3:**
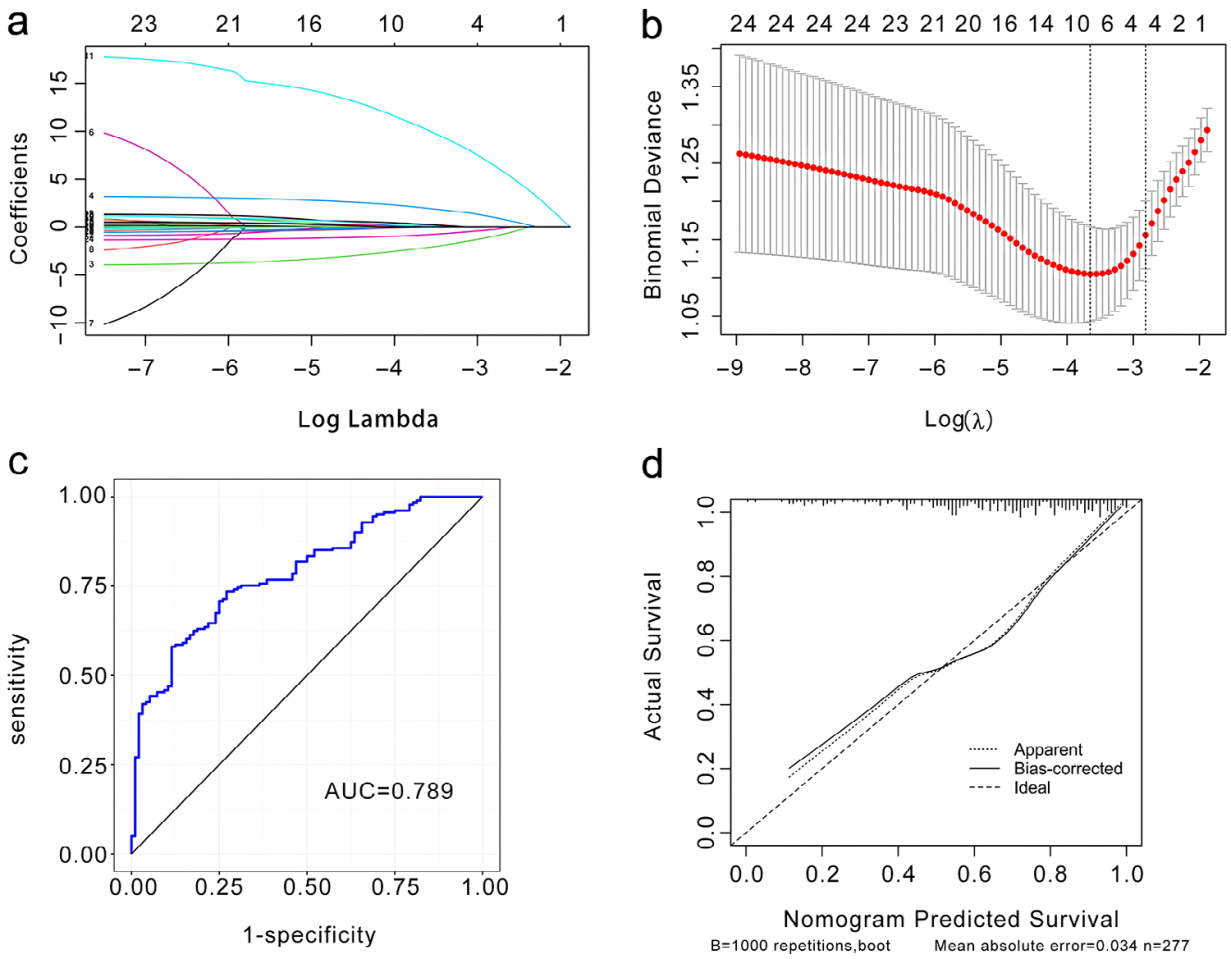
LASSO regression model for screening factors affecting improvement in NIHSS scores. (a) Path diagram of regression coefficients. (b) Cross‐validation curve. (c) Curve of subjects' work characteristics. (d) Calibration curve.

This study included 25 factors affecting the improvement of NIHSS scores. Considering the potential multicollinearity among the variables, LASSO regression was used to reduce variable dimensionality. The resultant path of regression coefficients was plotted (Figure 3a), and cross‐validation curves were drawn (Figure 3b). Based on the clinical context, lambda.1se was used as the optimal value of the model, and four variables were included in the logistic regression model (Table 2). These variables—namely, disease duration, treatment duration, AF, and NLR reduction values—emerged as predictors and influencers of neurological function recovery after cerebral infarction. Validation via the ROC curve yielded an area under the curve (AUC) value of 0.789 for this prediction model (Figure 3c). Then, the curve was calibrated as shown by the horizontal and vertical coordinates in Figure 3d, revealing that the predicted rate of neurological function recovery was 0.1–1.0. This aligns closely with the actual incidence (0.2–1.0), and the actual recovery rate was slightly higher. Although the apparent (internal correction) and bias‐corrected (external correction) were not completely fitted to the diagonal line (the actual probability of incidence = the predicted probability of incidence), the average absolute error was 0.034, suggesting that the model has good accuracy and predictive consistency.

**TABLE 2:** Logistic regression model for the NIHSS score improvement.

Intercept and variables	Amount	*β*	SE	Wald	*p*	OR	95% CI
Intercept		0.250	0.513	0.49	0.626	1.284	0.769–2.143
Disease duration		−0.111	0.032	−3.48	0.000*	0.895	0.866–0.924
Treatment duration		0.068	0.021	3.24	0.001^#^	1.071	1.048–1.094
NLR reduction values		0.735	0.142	5.18	< 0.0001*	2.086	1.810–2.404
Atrial fibrillation							
No	243	−1.342	0.453	−2.96	0.003^#^	0.261	0.166–0.411
Yes	34						

Abbreviation: NLR, neutrophil‐to‐lymphocyte ratio.

Statistically significant covariates: *p < 0.001, ^#^ *p* < 0.01.

### 3.2 Comparison of the Effectiveness Between Patients in High‐Efficacy and Low‐Efficacy Groups Before and After Treatment

As shown in the logistic regression model in Table 2, disease duration, treatment duration, AF, and NLR reduction value were factors influencing neurological function recovery, with NLR reduction value having the smallest *p* value (*p* < 0.0001). To further validate the relationship between NLR reduction value and neurological function recovery after cerebral infarction, we examined changes in NIHSS score and NLR before and after treatment in the high‐efficacy group (NIHSS score reduction ≥ 18%) and the low‐efficacy group (NIHSS score reduction or increase < 18%).

#### 3.2.1 Comparison of Baseline Information Before and After Propensity Matching

Table 3 illustrates a significant difference in sample sizes between both groups. Additionally, disease duration, treatment duration, admission NIHSS score, admission lower extremity muscle strength score, and admission NLR before propensity matching were found to be incongruous (*p* < 0.05). To ensure the comparability of baseline data between the groups, 1:1 propensity score matching was performed on patients' sex, age, disease duration, treatment duration, admission NIHSS score, admission upper and lower extremity muscle strength score, and admission NLR (with a tolerance value of 0.03). The final matching yielded 79 cases. After matching, the balance between the groups was comparable (all *p* > 0.05, Table 3).

**TABLE 3:** Comparison of the pretreatment baseline data.

Baseline data	Pre‐matching		After matching			
	High‐efficacy (*n *= 181)	Low‐efficacy (*n* = 96)	*p*	High‐efficacy (*n* = 79)	Low‐efficacy (*n* = 79)	*p*
Sex, *n* (%)^a^						
Female	48 (26.5)	21 (21.9)	0.395	16 (20.3)	16 (20.3)	1.000
Male	133 (73.5)	75 (78.1)		63 (79.7)	63 (79.7)	
Age (year)^b^	63 (55, 67.5)	61.5 (55, 68.75)	0.703	64 (55, 69)	61 (55, 69)	0.497
Disease duration^b^	10 (8, 13)	10.5 (9, 14)	0.018^&^	11 (9, 14)	10 (9, 13)	0.557
Treatment duration^b^	19 (14, 26.5)	14 (14, 18)	0.000*	15 (14, 21)	14 (14, 19)	0.255
NIHSS^b^	7 (5, 10)	6 (4, 9)	0.014^&^	6 (4, 10)	6 (4, 9)	0.879
UEMSS^b^	2 (1, 5)	2 (1, 3)	0.263	2 (1, 4)	2 (1, 3)	0.736
LEMSS^b^	2 (1, 3)	2 (1, 2)	0.008^#^	2 (1, 2)	2 (1, 2)	0.987
NLR^b^	3.22 (2.41, 4.60)	2.68 (2.02, 3.57)	0.000*	2.88 (2.16, 3.96)	2.88 (2.16, 3.82)	0.988

*Note*: NIHSS, UEMSS, LEMSS, and NLR are all at admission. Statistically significant covariates: For between‐group comparisons, *p* > 0.05 suggests the baseline concordance, *p < 0.001, ^#^ *p* < 0.01, ^&^ *p* < 0.05.

Abbreviations: IQR, interquartile range; LEMSS, lower extremity muscle strength score; NIHSS, National Institutes of Health Stroke Scale; NLR, neutrophil‐to‐lymphocyte ratio; UEMSS, upper extremity muscle strength score.

^a^ Data are n (%), *χ* ^2^ test.

^b^ Median (IQR), Mann–Whitney *U* test.

#### 3.2.2 Comparison of the Efficacy of the Two Groups of Patients Before and After Treatment

##### 3.2.2.1 Comparison of the NIHSS Scores, NLR, and Their Reduced Values Before and After Treatment in the Two Patient Groups

Tables 4 and 6 show the within‐group comparisons after treatment, revealing significant improvements in NIHSS scores (both *p* < 0.001) and NLR p<0.001 in the high‐efficacy group and p<0.01 in the low‐efficacy group) for patients in both groups. In terms of between‐group comparisons after treatment, the high‐efficacy group demonstrated a significant improvement in NIHSS scores (*p* < 0.001) and the NLR (*p* < 0.01) compared with the low‐efficacy group. Additionally, the NIHSS reduction scores (*p* < 0.001) and NLR reduction values (*p* < 0.05) were more significant in the high‐efficacy group with better efficacy, suggesting consistency between the two values before and after treatment. The high‐efficacy groups’ improved NIHSS scores were associated with a greater reduction in NLR, suggesting a consistent dynamic pattern of change before and after treatment for both values.

**TABLE 4:** Comparison of the NIHSS scores and NLR before and after treatment between the two groups.

Groups	NIHSS		NLR	
	Pretreatment	Posttreatment	Pretreatment	Posttreatment
High‐efficacy (*n* = 79)	6 (4, 10)	4 (2, 7)^a^*^,^ ^b^ ^★^	2.88 (2.16, 3.96)	2.03 (1.62, 2.69)^a^*^,^ ^b^ ^◆^
Low‐efficacy (*n* = 79)	6 (4, 9)	6 (4, 9)^a^*	2.88 (2.16, 3.82)	2.61 (1.84, 3.45)^a^

*Note*: Statistically significant covariates: Comparison within group after treatment, *p < 0.001,P<0.01; comparison with the low‐efficacy group after treatment, ^★^ *p* < 0.001, ^◆^ *p* < 0.01.

Abbreviations: IQR, interquartile range; NIHSS, National Institutes of Health Stroke Scale; NLR, neutrophil‐to‐lymphocyte ratio.

^a^ Comparison within group after treatment, Wilcoxon signed‐rank test, data are median (IQR).

^b^ Comparison with the low‐efficacy group after treatment, Mann–Whitney *U* test, data are median (IQR).

##### 3.2.2.2 Comparison of the Upper and Lower Extremity Muscle Strength Scores and Their Reduction Values Before and After Treatment in the Two Patient Groups

As demonstrated in Tables 5 and 6, the within‐group analysis revealed that both groups exhibited significant reductions in upper (p＜0.001 in the high‐efficacy group and p＜0.01 in the low‐efficacy group) and lower (*p* < 0.001 in the high‐efficacy group and *p* < 0.05 in the low‐efficiency group) extremity muscle strength scores following treatment. Upon between‐group comparison after treatment, the high‐efficacy group exhibited a more significant improvement in upper (*p* < 0.05) and lower (*p* < 0.01) limb muscle strength scores compared to the low‐efficacy group. The values of reduction in upper and lower extremity muscle strength scores before and after treatment were more significant in the high‐efficacy group (*p* < 0.001).

**TABLE 5:** Comparison of the muscle strength scores between the two groups before and after treatment.

Groups	UEMSS		LEMSS	
	Pretreatment	Posttreatment	Pretreatment	Posttreatment
High‐efficacy (*n* = 79)	2 (1, 4)	2 (1, 3)^a^*^,^ ^b^ ^▲^	2 (1, 2)	1 (1, 2)^a^*^,^ ^b^ ^◆^
Low‐efficacy (*n* = 79)	2 (1, 3)	2 (1, 3)^a^	2 (1, 2)	2 (1, 2)^a^

*Note*: Statistically significant covariates: Comparison within group after treatment, *p < 0.001, ^#^ *p* < 0.01, P<0.05; comparison with the low‐efficacy group after treatment, ^◆^ *p* < 0.01, ^▲^ *p* < 0.05.

Abbreviations: IQR, interquartile range; LEMSS, lower extremity muscle strength score; UEMSS, upper extremity muscle strength score.

^a^ Comparison within group after treatment, Wilcoxon signed‐rank test, data are median (IQR).

^b^ Comparison with the low‐efficacy group after treatment, Mann–Whitney *U* test, data are median (IQR).

**TABLE 6:** Comparison of the reduced values before and after treatment in the two groups.

Groups	Reduction values			
	NIHSS	UEMSS	LEMSS	NLR
High‐efficacy (*n* = 79)	2 (1, 3)^a^*	0 (0, 1)^a^*	0 (0, 1)^a^*	0.73 (0.15, 1.6)^a^ ^&^
Low‐efficacy (*n* = 79)	0 (0, 1)	0 (0, 0)	0 (0, 0)	0.29 (−0.34, 1.02)

*Note*: Statistically significant covariates: Comparison with the low‐efficacy group after treatment, *p < 0.001, ^&^ *p *< 0.05.

Abbreviations: IQR, interquartile range; LEMSS, lower extremity muscle strength score; NIHSS, National Institutes of Health Stroke Scale; NLR, neutrophil‐to‐lymphocyte ratio; UEMSS, upper extremity muscle strength score.

^a^ Comparison with the low‐efficacy group after treatment, Mann–Whitney *U* test, data are median (IQR).

### 3.3 Linear Correlation

The consistent fluctuations in NLR and brain NIHSS scores between the two groups, pretreatment and posttreatment, reaffirm the potential presence of a specific correlation between NLR and NIHSS scores. A bivariate correlation analysis was conducted to analyze the association between the NLR reduction values and NIHSS score reduction values, as well as the rate of NIHSS score reduction before and after treatment in 277 patients. The results showed that the correlation between the NLR reduction value and the NIHSS score reduction value, as well as the rate of NIHSS score reduction, were all statistically different (*p* < 0.001), and the dynamic change of NLR and NIHSS scores was positively correlated according to the *R* value (Table 7).

**TABLE 7:** Correlation between the NLR reduction values, NIHSS score reduction values, and NIHSS score reduction rate.

NLR reduction values	NIHSS reduction values	NIHSS reduction rate
*R* value	0.327	0.318
*p* value	0.000^a^*	0.000^a^*

*Note*: Statistically significant covariates: *p < 0.001.

Abbreviations: NIHSS, National Institutes of Health Stroke Scale; NLR, neutrophil‐to‐lymphocyte ratio.

^a^ bivariate analysis.

### 3.4 Predictive Value of NLR for Neurological Recovery by ROC Curve Analysis

The study findings revealed a positive correlation between the NLR reduction value and both the NIHSS reduction value and the NIHSS score reduction rate before and after treatment. To determine the independent predictive effect of NLR reduction on neurological recovery, the continuous variable NLR reduction value was used as the independent variable. Simultaneously, the dependent variable was defined as whether the reduction rate in NIHSS scores was ≥ 18%, serving as a measure of neurological recovery efficacy. Then, binary one‐way logistic regression was performed, and ROC curves were used to ascertain the cutoff value of the NLR reduction value for predicting a neurological functional recovery in patients with AIS.

#### 3.4.1 Binary One‐Factor Logistic Regression Analysis

The outcome of binary logistic regression analysis suggested that the NLR reduction value had a significant effect on neurological recovery (*p* = 0.000 < 0.001, Table 8), and the diagnostic predictive value of the NLR reduction in neurological recovery was further analyzed using ROC curves.

**TABLE 8:** One‐way logistic regression of the effect of reduced values of NLR on neurological recovery.

	*B*	SE	Wald	*p*	OR	95% CI
NLR reduction values	0.690	0.130	27.996	0.000*	1.994	1.544–2.574

*Note*: Statistically significant covariates: *p < 0.001.

Abbreviation: NLR, neutrophil‐to‐lymphocyte ratio.

#### 3.4.2 ROC Curves of NLR Reduction Values Before and After Treatment for Predicting a Neurological Recovery in Patients With AIS

**FIGURE 4:**
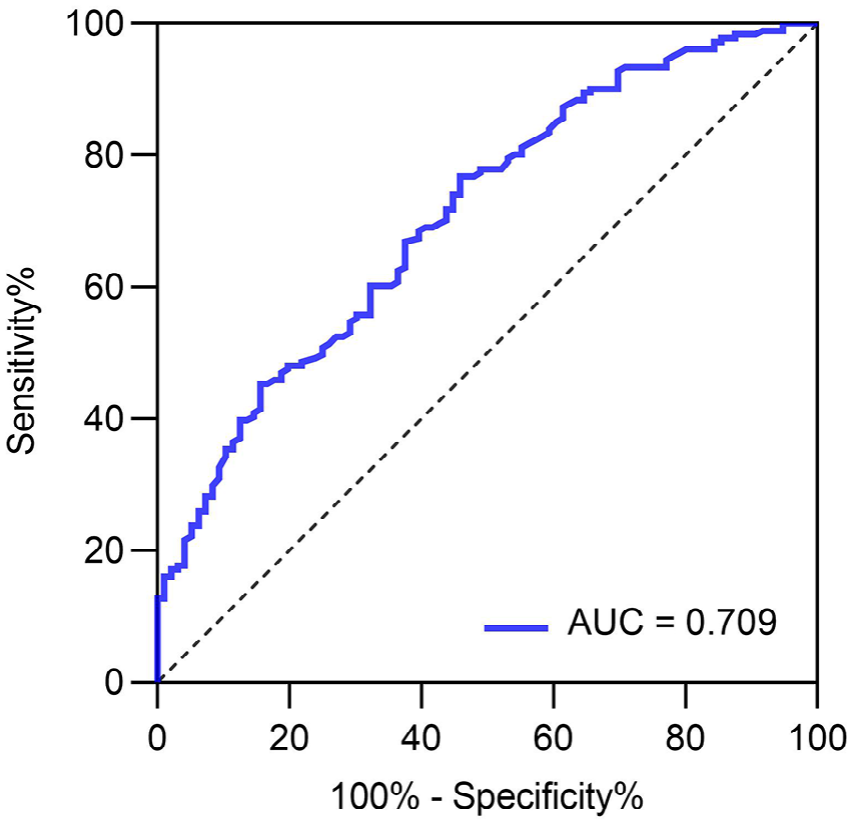
ROC curves for NLR reduction values to predict neurological recovery in AIS patients.

The results of the ROC curve analysis indicated that the AUC of NLR for predicting a neurological recovery in noninfected patients with acute cerebral infarction after thrombolysis was 0.709 (95% CI: 0.649–0.772, *p* < 0.001). Its optimal cutoff value, identified at the maximum value of Youden's index of 0.31, was 0.335, with a sensitivity of 76.8% and a specificity of 54.2% (Figure 4).

The results of the ROC curve analysis revealed that the cutoff value of the NLR reduction value for predicting a neurological recovery in noninfected patients with acute cerebral infarction after thrombolysis was 0.335 and that neurological recovery was good when the NLR reduction value was ≥ 0.335 after treatment and poor when the NLR reduction value was < 0.335 after treatment.

### 3.5 Binary Logistic Regression of NLR Reduction Values to Predict Neurological Recovery in AIS Patients

To mitigate the influence of clinically relevant confounding factors, we used the NIHSS score reduction rate of ≥ 18% as the outcome variable to determine neurological improvement. The patients were classified according to whether the NLR reduction value of ≥0.335 after treatment, and the influencing factors obtained from the LASSO regression (disease duration, treatment duration, and AF) were matched by 1:1 propensity scores between the groups. One‐way logistic regression of NLR reduction values was performed again, excluding the interference of these factors, to verify the independent predictive effect of NLR reduction values on neurologic recovery.

#### 3.5.1 Comparison of Relevant Impact Factors Before and After Propensity Matching

To mitigate the influence of confounding factors such as disease duration, treatment duration, and AF, we subjected these variables to 1:1 propensity matching between groups with NLR reduction values was ≥ 0.335 and groups with NLR reduction values of < 0.335, with the tolerance value set at 0.03. The final matching yielded 93 cases, with 1 complete match and 92 fuzzy matches. The difference in disease duration (*p* < 0.01) and treatment duration (*p* < 0.05) before treatment was statistically significant, and there was no difference in disease duration, treatment duration, or AF between groups after matching (*p* > 0.05), Table 9.

**TABLE 9:** Baseline comparison of the impact factors.

Influencing factors	Pre‐matching			After matching		
	≥ 0.335 (*n* = 183)	< 0.335 (*n* = 94)	*p*	≥ 0.335 (*n* = 93)	< 0.335 (*n *= 93)	*p*
Disease duration^b^	11 (9, 14)	9 (8, 12)	0.007^#^	10 (8, 12.5)	9 (8, 12)	0.467
Treatment duration^b^	16 (14, 26)	14 (14, 20)	0.016^&^	15 (14, 21)	14 (14, 20)	0.711
Atrial fibrillation^a^	22 (12.0)	12 (12.8)	0.858	13 (14.0)	12 (12.9)	0.830

*Note*: Statistically significant covariates: for between‐group comparisons, *p *> 0.05 suggests baseline concordance, ^#^ *p *< 0.01, ^&^ *p* < 0.05.

Abbreviation: IQR, interquartile range.

^a^ Data are n (%), *χ* test.

^b^ median (IQR), Mann–Whitney *U* test.

#### 3.5.2 One‐Factor Logistic Regression Analysis After Propensity Matching

A matched post hoc logistic regression analysis was performed to examine the relationship between the NLR reduction value (whether of ≥ 0.335) as the independent variable and the NIHSS score reduction rate (whether of ≥ 18%) as the dependent variable. The results demonstrated that after excluding the interference of relevant confounders, patients with NLR reduction values ≥ 0.335 exhibited a good neurological recovery, which was 4.348 times higher than that of patients with NLR reduction values < 0.335 (*p* = 0.000 < 0.001, Table 10). Combined with the results of ROC curve analysis, it suggests that the posttreatment NLR reduction value is an independent predictor of neurological function recovery in noninfected patients with acute cerebral infarction after thrombolysis. Moreover, a larger NLR reduction value suggests a more favorable prognosis for neurological function after treatment.

**TABLE 10:** One‐way logistic regression of the effect of NLR reduction values on neurological recovery.

	*B*	SE	Wald	*p*	OR	95% CI
NLR reduction value (< 0.335)	1.470	0.324	20.550	0.000*	4.348	2.303–8.210

*Note*: Statistically significant covariates: *p < 0.001.

Abbreviation: NLR, neutrophil‐to‐lymphocyte ratio.

## 4 Discussion

GBD 2019 Stroke Collaborators (2021) showed that the annual number of strokes and deaths due to stroke increased substantially from 1990 to 2019, with one‐third of these patients left with a disability (Brodtmann and van de Port, 2013). Stroke is the leading cause of death in China, and a large cross‐sectional study of the stroke burden in China suggests that the estimated prevalence, incidence, and mortality rate of stroke in 2020 were 2.6%, 505.2 per 100,000 person‐years, and 343.4 per 100,000 person‐years, respectively, indicating the need for an improved stroke prevention strategy in the general Chinese population (Tu et al., 2023). Cerebral infarction accounts for 80% of strokes (Mozaffarian et al., 2016). The primary treatment recommended by current guidelines for AIS is intravenous thrombolysis or endovascular thrombolysis within a specific time window. The pathogenesis of AIS is complex, and the inflammatory response plays an important role in postischemic reperfusion injury (Shi et al., 2019). Identifying effective prognostic predictors is vital for clinical diagnosis and treatment.

Leukocytes, integral components of immunity and inflammation (Xie et al., 2023), include neutrophils, the most important leukocyte subtype. Neutrophils are recruited into the ischemic region of the brain at the early stage of inflammation. On the one hand, this contributes to secondary brain damage in the ischemic hemidiaphragm region through the release of oxygen‐free radicals (Ceulemans et al., 2010). On the other hand, as an important source of matrix metalloproteinase‐9, they can cause hemorrhagic transformation (HT) by disrupting the blood–brain barrier through the release of cytokines, chemokines, and adhesion factors (Ying et al., 2020). Specific subtypes of lymphocytes, regulatory T cells, are the main cerebroprotective immunomodulators in acute cerebral infarction, reducing infarct size and ameliorating neurological deficits by eliminating the inflammatory response (Liesz et al., 2013; Gong et al., 2019). As a biocomplex marker that responds to both neutrophil inflammatory infiltration and lymphocyte cerebroprotective modulation, NLR potentially outperforms other leukocyte subtypes and their dynamic changes, as well as individual biomarkers, in predicting neurological deterioration in the early post‐thrombolytic period in patients with AIS (Xie et al., 2023; Gong et al., 2019). Elevated NLR after mechanical thrombectomy or r‐tPA treatment is a significant predictor of HT, early neurologic dysfunction, morbidity, and mortality (Xie et al., 2023), and high NLR levels are associated with increased infarct size (Gong et al., 2019). Current research mainly focuses on the NLR prediction of neurologic recovery after thrombolysis within baseline optimal prediction ranges. Studies have shown that the optimal cutoff value for NLR in predicting early neurologic deterioration in patients after intravenous thrombolysis is 4.43 (Gong et al., 2019), the cutoff value for predicting the risk of HT in patients with AIS is 4.96 (R. Zhang and Chu, 2022), and the diagnostic cutoff value for NLR levels upon admission for predicting a poor prognosis in patients with AIS is 2.84 (Zhai et al., 2017). The specific predictive value of the NLR varies across studies. In addition to exploring the predictive value of NLR at baseline, some studies have also investigated the predictive value of dynamic changes in NLR before and after treatment. The NLR, as a dynamic variable, exhibits a higher predictive value after intravenous thrombolysis (Guo et al., 2016). Ying et al. (Ying et al., 2020) observed dynamic changes in NLR at admission, 24 h, and 7 days post r‐tPA infusion. They found that a significant increase in the NLR at 24 h and Day 7 after thrombolysis was associated with a poor 3‐month functional prognosis. Another related study found positive correlations between dynamic changes in the NLR after intravenous thrombolysis in patients with AIS and changes in NIHSS scores, which determined the degree of neurological damage in cerebral infarction (Komurcu et al., 2020). Therefore, it is critical to understand the impact of changes in NLR on patient prognosis. However, little has been reported about the predictive role of values that decrease during dynamic changes in NLR, whether exploring the predictive value of baseline NLR or dynamic changes in NLR. In addition, most of these studies did not discuss infected and noninfected patients separately, and there was some confounding interference. Yet, relevant studies have shown that previous infections, hospital‐acquired infections in the early stages of stroke, and elevated leukocyte counts are all associated with poor prognosis (Guo et al., 2016). In this study, we limited these potential confounding factors by excluding infected patients. We only discussed the effect of changes in NLR reduction values pretreatment and posttreatment on noninfected patients after thrombolysis. We also employed LASSO regression to identify NIHSS‐related influencing variables before logistic regression. To control the effects of these confounding variables, propensity matching was employed, enhancing the accuracy of the predictive value of the NLR reduction values.

In this study, a predictive model of neurological recovery was established by screening the values of disease duration, treatment duration, AF, and posttreatment NLR reduction through LASSO regression. Risk factors included increased disease duration and AF, whereas protective factors included prolonged treatment duration and increased NLR reduction value after treatment. At present, the Xingnao Kaiqiao acupuncture is mainly devoted to the study of the manipulative quantitative effect of acupuncture, that is, the correlation study between the acupuncture manipulation and the clinical effect, in which the time–effect relationship of acupuncture is one of the important contents of the quantitative study of acupuncture manipulation (Shi, 2012; Y. Li, 2013). In the time–effect relationship of acupuncture, in addition to the duration of needle retention per treatment and the frequency of acupuncture (Shi, 2012; Y. Li, 2013), the time of the start of the acupuncture intervention (R. Li, Wang, et al., 2021; Chen et al., 2024; Zhuo et al., 2021) as well as the total length of time that the acupuncture intervention lasts (Y. Li, 2013; X. Zhang et al., 2019) is also closely related to the efficacy of acupuncture. Previous studies have demonstrated that the earlier the acupuncture intervention is initiated after cerebral infarction, the better the late recovery is (R. Li, Wang et al., 2021; Chen et al., 2024; Zhuo et al., 2021) and that patients with a treatment course of 28 days exhibited more significant NIHSS score improvements than those with a treatment course of 14 days (X. Zhang et al., 2019). Table 1 suggests that patients with better recovery had a shorter disease duration, and most of them had a treatment course of more than 14 days, which is consistent with the results of previous studies. The patients in this study were the first time to receive acupuncture treatment after cerebral infarction, and the shorter the duration of the disease indicated that the earlier the acupuncture intervention was started, which also indicates the importance of early acupuncture intervention and long‐term acupuncture intervention. Xingnao Kaiqiao acupuncture treatment has been widely promoted in major Chinese medicine hospitals across the country. Regardless of whether thrombolysis, its effectiveness and safety for rehabilitation after cerebral infarction have been confirmed by multicenter, large‐sample randomized control trials (S. Zhang et al., 2015; Xian et al., 2022; Wang et al., 2017; Q. Zhang and Tian, 2020; Song et al., 2022). Currently, the hotspots of the research on the Xingnao Kaiqiao acupuncture method mainly focus on early acupuncture intervention. However, most of the patients were treated in Western medicine hospitals within 1 week after cerebral infarction, and most of these patients were transferred to Western medicine hospitals for treatment at a later stage, which led to the interruption of acupuncture intervention. Thus, this is the main goal of our later work: to extend the brain‐awakening acupuncture method to Western medicine hospitals and to provide patients with acupuncture interventions as soon as the treatment begins, with the aim of providing timely and earlier acupuncture interventions for patients with cerebral infarction and facilitating the implementation of long‐term acupuncture rehabilitation.

AF, an important risk factor for the prognosis of cerebral infarction (Hankey, 2017), also has a bidirectional relationship with NLR, which is both independently predictive of and affected by AF (Chang et al., 2023; Saliba et al., 2015) and influences neurological recovery synergistically. The chronic disease state of patients with cardiovascular risk leads to the release of granulocyte‐like myeloid‐derived suppressor cells in the bone marrow that inhibit lymphocytes, contributing to an increased NLR and, consequently, a higher baseline NLR in patients with AF compared to those with non‐AF (Chang et al., 2023). A high NLR is independently associated with an increased risk of stroke in AF (Saliba et al., 2015). Thus, active control of AF is important for recovery after cerebral infarction.

Numerous studies have demonstrated the effectiveness of Xingnao Kaiqiao acupuncture, a potent stimulating acupuncture therapy when used in conjunction with standard treatment in Western medicine. It has consistently been reported to inhibit the inflammatory response and improve both motor and neurological functions in patients with cerebral infarction when combined with two therapies (Xian et al., 2022; Wang et al., 2017). Administering Xingnao Kaiqiao acupuncture after intravenous thrombolysis in patients with AIS effectively promotes the restoration of neurological impairments (Q. Zhang and Tian, 2020; Song et al., 2022). The findings of intragroup and intergroup comparisons of pretreatment and posttreatment in the high‐ and low‐efficacy groups revealed that comprehensive acupuncture intervention reduced NIHSS scores and muscle strength scores of the affected limbs of patients with cerebral infarction who underwent intravenous thrombolysis with r‐tPA and effectively enhanced both neurological and motor functions in patients after treatment. The NLR values of the high‐efficacy group with better recovery decreased more after treatment, suggesting a link between NLR reduction values before and after treatment and neurological recovery. Bivariate correlation analyses of NLR reduction values with NIHSS score reduction values and the rate of NIHSS score reduction were all statistically significant (*p* < 0.001), indicating a positive correlation between NLR reduction values and neurological recovery, consistent with prior research (Komurcu et al., 2020) and further reinforcing the notion that an increased NLR reduction value before and after treatment is a protective factor for neurological recovery. To further assess the optimal predictive significance of posttreatment NLR reduction on neurological recovery (using a ≥ 18% reduction in NIHSS score as the efficacy benchmark) in ROC curve analysis, this study initially performed binary one‐way logistic regression. The findings indicate that the NLR reduction value has a significant impact on neurological recovery (*p* < 0.001). The ROC curve results suggest 0.335 as the critical threshold for the NLR reduction value in predicting neurological recovery. The neurological recovery of noninfected patients with AIS was better when the NLR reduction value was ≥ 0.335, and the accuracy of diagnosis was 70.9%. Additionally, to eliminate the impact of disease duration, treatment duration, and AF on neurological recovery, the independent predictive significance of NLR reduction values pretreatment and posttreatment was further established to substantiate the precision of NLR reduction values in predicting the threshold values of ROC curves for neurological recovery in patients with AIS. In this study, the grouping was based on whether the NLR reduction value was ≥ 0.335 before and after treatment, and the matching variables used were disease duration, treatment duration, and AF, which were identified as influencing factors of neurological recovery via LASSO regression. To eliminate interference from these factors, a 1:1 propensity score matching was conducted between the groups, followed by a one‐way logistic regression analysis. The findings revealed that the NLR reduction value independently predicted neurological recovery after cerebral infarction. Patients with an NLR reduction value of ≥ 0.335 exhibited 4.348 times higher efficacy in neurological recovery than patients with an NLR reduction value of < 0.335, while higher levels of NLR reduction indicate a more favorable prognosis for neurological function following treatment.

Studies have shown that acupuncture (Liu et al., 2020; N. Li, Guo et al., 2021), moxibustion (Yang et al., 2024; Yang et al., 2024), and herbs with heat and toxin removal effects (He et al., 2024; Feng et al., 2025) have the efficacy of regulating immunity and inhibiting inflammation, so this close attention to the dynamic changes in the NLR before and after the treatment of the patient, and the early identification of the changes in the inflammatory status of the patient's body is clinically important for guiding the early Chinese medicine‐assisted anti‐inflammatory treatment. All patients in this study were transferred to our hospital to continue treatment after thrombolysis in Huanhu Hospital. In the future, for newly admitted patients, the preadmission NLR can be calculated based on the results of their complete blood count tested at the previous hospital, and then the first complete blood count tested after admission can be subtracted from the NLR obtained from the preadmission NLR. If the NLR did not decrease but rather increased or if the NLR reduction was lower than 0.335, then under the antibiotic rational use, adjunctive anti‐inflammatory therapy such as acupuncture, moxibustion, and herbal medicine can be administered. For patients transferred from outside hospitals or not, in order to better understand the change of NLR after a period of treatment, a complete blood count can be retested at the time of discharge, and the NLR after treatment can be subtracted from the admission NLR. If the NLR reduction value is suggested to be less than 0.335, or the NLR is increased after treatment, considering the poor prognosis that inflammation may potentially affect, the patient can be advised to continue anti‐inflammatory and TCM adjuvant therapy to enhance immunity in a comprehensive outpatient clinic after discharge with a view to improving the patient's late recovery.

In this study, although the ROC curve analysis yielded an AUC of 0.709 for the NLR reduction value as a predictor of neurological recovery, a substantial correlation between NLR and neurological recovery was evident via LASSO regression analysis. The consistent pattern of NLR and NIHSS score fluctuations pretreatment and posttreatment, the correlation between NLR reduction values and NIHSS scores, and the incorporation of NLR reduction in the logistic regression model collectively suggest that variations in peripheral blood NLR can serve as an indicator for initially assessing the prognosis of noninfected patients with cerebral infarction. The slightly lower predictive value may be associated with the study's relatively limited sample size and its exclusive focus on data obtained from patients who underwent thrombolysis for cerebral infarction within a month of disease onset during their hospitalization. Moreover, inadequate follow‐up during subsequent treatment phases hindered a comprehensive investigation into the long‐term effectiveness of acupuncture combined therapy for cerebral infarction. In addition, due to the small sample size of infected patients, it was not possible to fully analyze the relationship between changes in NLR dynamics and neurological recovery in the infected population. Future studies should encompass a broader range of patients, including those who did not receive thrombolysis treatment and those with non‐first stroke occurrences, in order to expand the sample size, as well as analyze a separate cohort of infected patients. This would help to explore and validate the predictive value of reduced NLR values for neurologic recovery in the nonthrombolytic population and in the infected population on a larger scale. In addition, post‐discharge patients should be followed up with inflammatory biomarker levels over more time periods to understand the relationship between changes in biomarker levels over time and neurologic recovery. A prospective analysis of the long‐term efficacy of the treatment will offer guidance for decision‐making and predicting a functional recovery in the clinical management of ischemic stroke.

## 5 Conclusion

In summary, the amalgamation of Xingnao Kaiqiao acupuncture and fundamental therapy rooted in the Western tradition can improve neurological deficits and motor function in noninfected patients with AIS who have undergone intravenous thrombolysis with r‐tPA. Additionally, the pretreatment and posttreatment reduction of NLR values showed a positive relationship with neurological function recovery following cerebral infarction. Furthermore, the NLR reduction value functions as an independent predictor, while higher levels of NLR reduction indicate a more favorable prognosis for neurological function following treatment. Therefore, paying close attention to the dynamic changes of NLR before and after the treatment of patients with cerebral infarction, and under the reasonable use of antibiotics, early and long‐term acupuncture intervention can help to inhibit inflammation in the body of patients and promote the recovery of their neurological function.

## Author Contributions

**Yiran Zhao**: writing–review and editing, writing–original draft, data curation, methodology, conceptualization. **Xu Wang**: writing–review and editing, writing–original draft, methodology, conceptualization. **Wenxiu Qin**: writing–review and editing, investigation, data curation. **Shaojing Shi**: writing–review and editing, investigation, data curation. **Min Wang**: writing–review and editing, formal analysis, visualization. **Jinsheng Zhang**: writing–review and editing, supervision, validation. **Xin Zou**: writing–review and editing, supervision, validation. **Junfeng Xu**: writing–review and editing, methodology, conceptualization, project administration. **Jing Li**: writing–review and editing, methodology, conceptualization, project administration. **Xuemin Shi**: writing–review and editing, methodology, conceptualization.

## Ethics Statement

This study was approved by the Ethics Committee of the First Teaching Hospital of Tianjin University of Traditional Chinese Medicine (Approval No.: TYLL2020[K]057) and was registered in the Chinese Clinical Trial Registry (Registration No.: ChiCTR2100045415), and an informed consent was granted.

## Conflicts of Interest

The authors declare no conflicts of interest.

### Peer Review

The peer review history for this article is available at https://publons.com/publon/10.1002/brb3.70373.

## Data Availability

The datasets analyzed for the current study are not publicly available due to patient confidentiality and participant privacy restrictions but can be obtained from the corresponding author under certain conditions.
